# Peripheral magnetic stimulation effects on brain activity in older adults with schizophrenia – a randomized controlled pilot study

**DOI:** 10.3389/fpsyt.2026.1881961

**Published:** 2026-08-07

**Authors:** Yuntong Zhang, Yijun Chen, Roger Adams, Sophia Xenos, Zhengquan Chen, Xueming Cao, Bingfu Song, Jia Han

**Affiliations:** 1 College of Rehabilitation Sciences, Shanghai University of Medicine and Health Sciences, Shanghai, China; 2 Shanghai Yangpu District Mental Health Center, Shanghai, China; 3 Mental Health Center Affiliated to Shanghai University of Medicine and Health Sciences, Shanghai, China; 4 Research Institute for Sport and Exercise, University of Canberra, Canberra, ACT, Australia; 5 Applied Health, School of Health and Biomedical Sciences, RMIT University, Melbourne, VIC, Australia; 6 Changchun GeneScience Pharmaceuticals Co., Ltd, Changchun, China

**Keywords:** brain functional connectivity, older adults, peripheral magnetic stimulation, resting-state EEG, schizophrenia

## Abstract

**Objective:**

To conduct an exploratory neurophysiological pilot analysis of transient resting-state EEG alterations induced by a single peripheral magnetic stimulation (PMS) session in older adults with schizophrenia.

**Methods:**

In a randomized sham-controlled study, 40 older inpatients received either active PMS (30 minutes on the quadriceps) or a sham intervention. Eyes-closed resting-state EEG (59 channels) was recorded pre- and post-intervention. Spectral power (δ, θ, α, β, γ), scalp topographies, and weighted phase lag index (dwPLI) functional connectivity were analyzed.

**Results:**

Active PMS induced significant, frequency-specific changes: decreased δ/θ power and increased α/β/γ power. Topographically, low-frequency reductions were prominent in the midline-right hemisphere, while high-frequency enhancements occurred in frontoparietal regions. Connectivity analyses revealed a dual pattern: potentially strengthened long-range δ-θ coupling (frontal/midline to right posterior) and enhanced short-range high-frequency synchrony in frontal and lateral parietal areas. These dwPLI connectivity findings derive from exploratory analyses and were not prespecified formal study outcomes. The sham group showed no comparable changes.

**Conclusions:**

This exploratory pilot study identified transient band-specific resting-state EEG shifts following one PMS session, including reduced low-frequency oscillations and elevated high-frequency power and connectivity in older adults with schizophrenia. These preliminary neurophysiological patterns require replication in larger controlled trials; no definitive clinical therapeutic inferences can be drawn from this small exploratory dataset.

**Significance:**

This exploratory pilot analysis offers preliminary novel electrophysiological observations of PMS-related brain rhythm modulation in older adults with schizophrenia. These exploratory findings lay a foundation for future larger, confirmatory neurophysiological trials peripheral magnetic stimulation; schizophrenia; resting-state EEG; older adults.

**Clinical trial registration:**

## 1 Introduction

Schizophrenia is a complex, polygenic disorder that disrupts multiple domains of psychological functioning and is a leading cause of psychiatric disability (1, 2). According to the Global Burden of Disease 2019 study, an estimated 23.6 million people worldwide are affected (3). By 2050, the number of individuals aged ≥60 years living with schizophrenia is projected to exceed 10 million (4). Compared with younger cohorts, older adults with schizophrenia tend to exhibit less severe positive symptoms but more pronounced negative symptoms, including avolition, anhedonia, and blunted affect (5, 6). In later life, schizophrenia is also associated with premature mortality (7), a higher burden of somatic comorbidities (8), and a decline in quality of life and functional capacity (9). Despite substantial literature on schizophrenia, research specifically focused on older adults remains relatively limited—particularly regarding illness course, symptomatology, and treatment responsiveness (10).

Although the pathophysiological mechanisms of late-life schizophrenia remain unclear, cumulative evidence indicates that dysfunction of large-scale brain networks may be central to its symptomatology (11–13). Brain networks are highly dynamic, and electroencephalography (EEG) can capture state-dependent functional fluctuations at the systems level. In resting-state EEG (rsEEG), the spatial topography and spectral power of oscillatory activity display relatively stable patterns at rest but undergo marked modulation under external stimulation, revealing varying network responsiveness across different stimulus conditions (14, 15). As an indispensable tool in psychiatric research, rsEEG delineates baseline electrical activity in the absence of task demands and provides biomarkers for diagnosis and monitoring treatment effects, thereby enabling a deeper understanding of the dysregulated brain function and associated neural mechanisms in schizophrenia—and in particular among older adults (16).

A review by Newson et al. indicates that prior work has focused predominantly on resting-state alterations in lower-frequency bands (δ,<4 Hz;θ,4–7.5 Hz) and the mid-frequency α band (7.5–12.5 Hz), with comparatively less attention to higher-frequency β and γ rhythms (17). Relative to healthy adults, individuals with schizophrenia aged 60>? show significantly increased power in the low-frequency range (δ and θ) and markedly reduced power in the alpha band, whereas findings in the beta range (12.5–30 Hz) are less consistent overall, with some studies reporting a non-significant change (18). In a functional connectivity study, Di Lorenzo et al. found patients with schizophrenia (SCZ) had higher local phase synchrony (LPS) in the δ band, particularly between left frontal and right temporo-parietal, cingulate, and occipital regions. In theθband, those with SCZ showed elevated LPS across many regions but lower LPS between frontal, temporo-parietal, and cingulate scalp regions. The β1 sub-band showed hyperconnectivity, while β2 showed hypoconnectivity in patients with longer illness duration. In the gamma band, SCZ had higher LPS between right occipital, frontal, temporo-parietal, and cingulate regions (19). More recently, a study in older adults with schizophrenia found that the duration of microstate A (linked to visual processing) and D (associated with large-scale network integration) were significantly longer than in healthy older adults, while the occurrence of microstates A, B (executive and cognitive control), and C (affective processing) was reduced. These findings point to dysfunctional information processing—particularly in cognitive control and affective domains—and may help to explain the instability in large-scale network integration characteristic of schizophrenia in later life (20).

Most current treatment methods for schizophrenia focus on directly stimulating the central nervous system, such as repetitive transcranial magnetic stimulation (rTMS) and transcranial direct current stimulation (tDCS) (21, 22). Peripheral magnetic stimulation (PMS) has gained increasing attention in recent years as a non-invasive neuromodulation technique (23). PMS uses electromagnetic induction to generate currents in peripheral neuromuscular tissues, delivering relatively painless stimulation that can penetrate deeper conductive structures (24, 25). Stimulation of peripheral nerves by PMS engages feedback loops between the cerebral cortex and the peripheral nervous system, potentially modulating neural activity and circuits subserving affect, cognition, and motor control (23). Mechanistically, PMS elicits muscle contractions that send rich ascending, movement-related proprioceptive inputs to the brain and can alter primary motor cortex (M1) excitability via thalamo-cortical and spino-cerebellar pathways (26). A review by Diao et al. further indicates that PMS-induced currents can activate nerve terminals and be conveyed through neural pathways to the spinal cord and brain, helping to alleviate low back pain arising from nerve injury or compression (27).

To date, no studies have investigated the use of PMS for psychiatric disorders, particularly among older adults with schizophrenia. Although transcranial magnetic stimulation has been widely studied for schizophrenia, the efficacy and adverse effects of PMS in late-life schizophrenia remain to be determined. Accordingly, this study aims to explore the acute electrophysiological effects of PMS, an emerging neuromodulatory technique, in older adults with schizophrenia.

For the first time in this context, we employ resting-state electroencephalography (rsEEG) to characterize changes in brain activity following PMS, with a specific focus on the dynamics of neural oscillations and functional connectivity. Specifically, we construct scalp topographic maps and functional connectivity graphs before and after stimulation to quantify spatial changes in band-limited power across scalp regions and to examine network-level alterations in interregional coupling. We further analyze region-specific shifts in spectral power to explore the potential of PMS to facilitate functional recovery of brain networks. We hypothesize that PMS may modulate neural oscillations and interregional functional connectivity within brain networks in schizophrenia patients by modulating neural oscillations and enhancing functional connectivity between different brain regions, with these changes being reflected in resting-state electroencephalography (rsEEG).

## 2 Materials and methods

### 2.1 Design

This study employed a randomized, sham-controlled, two-arm pre—post-intervention design. Participants were assigned using block randomization with a block size of 4, at a 1:1 ratio to the active PMS group or sham group (20 participants per group). The random allocation sequence was independently generated by a professional statistician using R 4.2.3, who did not take part in participant recruitment or experimental operation. Allocation concealment was achieved by sequentially numbered, opaque, and sealed envelopes that were kept by a dedicated research coordinator and opened only after baseline assessment was completed. All eligible participants were screened and enrolled by two experienced psychiatrists who had no access to the random sequence and group information.

A triple-blind design was adopted in this study: participants, clinical assessors responsible for PANSS rating, EEG researchers conducting data preprocessing and analysis, and the statistician performing statistical analyses were all fully blinded to group assignment throughout the whole study.

**Figure 1:**
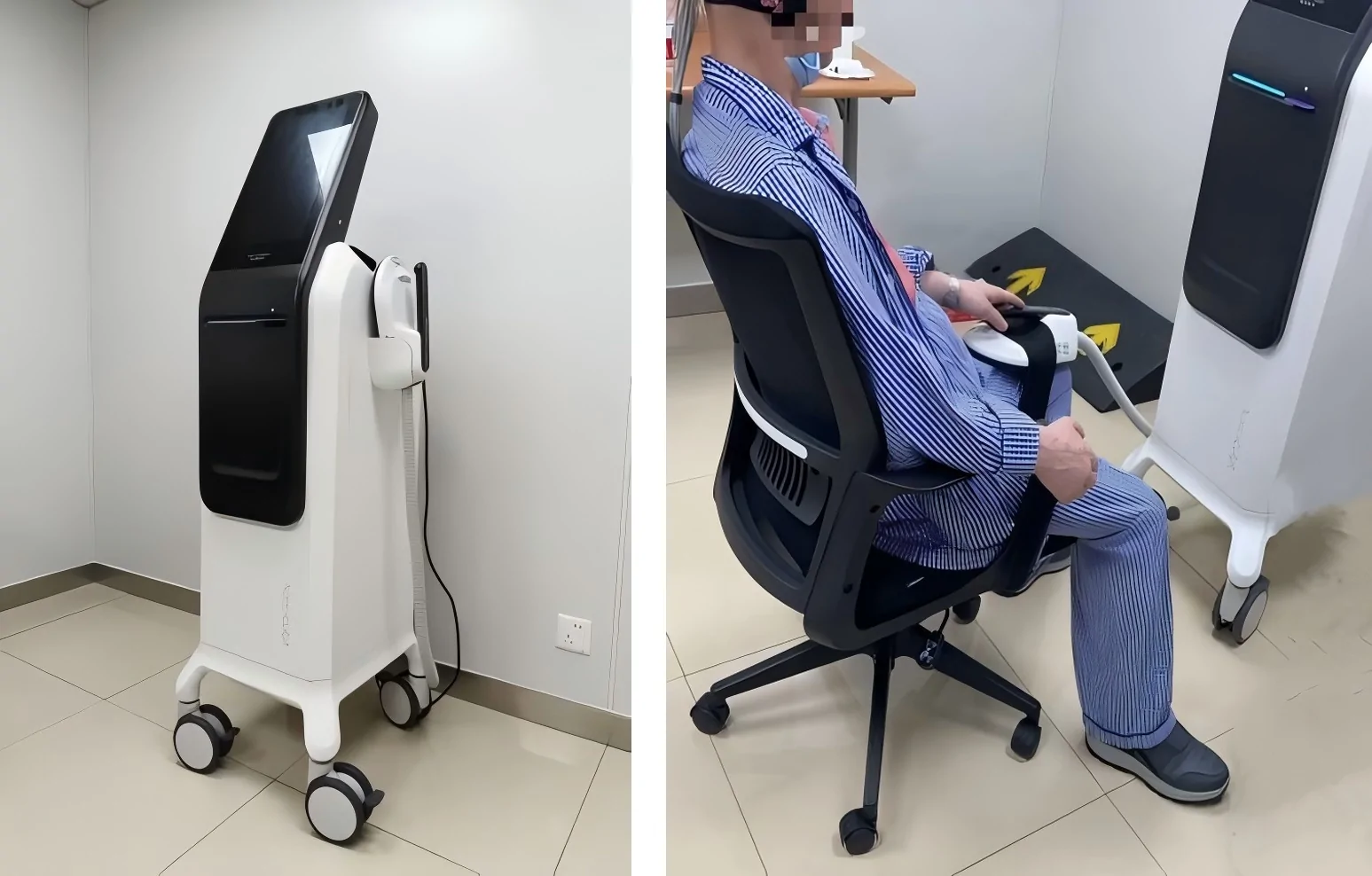
Peripheral magnetic stimulation (PMS) apparatus and standardized stimulation setup.

The PMS intervention was delivered using the peripheral magnetic stimulation device manufactured by Shanghai Weimei Medical Technology, as shown in **Figure 1**, consisting of a console driving a handheld circular coil that generates biphasic pulsed magnetic fields. The coil plane was positioned parallel to and in gentle contact with the skin, centered over the anterolateral thigh (femoral nerve projection area). The thigh was selected as the stimulation site to avoid the immediate cortical excitability confounds associated with direct transcranial stimulation while modulating central network activity via peripheral neuromuscular activation and somatosensory afferents ascending through spino–bulbar–cortical pathways.

Standardized stimulation parameters were: pulse frequency 10 Hz; duty cycle 5 s on/25 s off; single-session duration 30 min. The device’s rated peak magnetic flux density was ~1.8 T (coil-end charging voltage ~450 V). All sessions were conducted with participants seated and relaxed; metallic objects were removed beforehand, and the skin/coil positions were marked to ensure reproducible placement. Sham stimulation was implemented via coil decoupling, 90°coil tilt, and unloaded pulses, with acoustic and somatosensory cues fully matched to the active condition to guarantee blinding. All participants wore regular clothing during the procedure, so visual observation of underlying muscle contraction was not feasible. Most participants only reported mild superficial sensations without severe discomfort, and none could clearly differentiate active stimulation from sham stimulation. After intervention, participants were asked to guess their group assignment. The overall guessing accuracy was 57.5% (23 out of 40 participants), and a binomial test confirmed no significant difference from random guessing (p > 0.05), proving effective blinding in this study. Each arm included 20 participants (total N = 40). Baseline data were collected prior to the intervention, and outcome measures were obtained within 5 minutes after completing the stimulation session.

### 2.2 Participants

EEG data were obtained from 40 inpatients aged >60 years with a current diagnosis of schizophrenia who were recruited from the Department of Psychiatry, Yangpu District Mental Health Center, Shanghai, China. All participants met DSM-IV diagnostic criteria for schizophrenia, with diagnoses independently confirmed by two experienced psychiatrists. Eligibility required age ≥60 years and clinical stability without the need for acute psychiatric intervention.

Exclusion criteria included a history of neurological disorders (e.g., brain tumor, Parkinson’s disease, stroke, severe head injury), alcohol or drug abuse, and any contraindication to PMS or EEG recording. Written informed consent was obtained from all participants prior to performing study procedures. The study was approved by the Clinical Research Ethics Committee of Shanghai University of Medicine & Health Sciences (approval No. 202503650102197808083517). In addition, the study is registered on the ClinicalTrials.gov website in the United States with the registration number: NCT07194460.

### 2.3 EEG recording and preprocessing

Ten minutes of eyes-closed resting-state EEG (rsEEG) were recorded from the scalp in an electromagnetically shielded room. Signals were acquired with a 64-channel NeuSense W series wireless EEG system at a sampling rate of 1000 Hz. Preprocessing was performed in MATLAB R2021b (MathWorks, Natick, MA, USA) using the EEGLAB toolbox (28) as follows: (1) Non-scalp auxiliary channels (ECG; horizontal/vertical EOG: HEOR, HEOL, VEOU, VEOL) were excluded, leaving 59 scalp channels for analysis.(2) Data were band-pass filtered at 0.1–40 Hz; all filters were applied in a zero-phase, bidirectional manner. The data were then down-sampled to 500 Hz. (3) Bad channels were detected by the clean-rawdata plugin (criteria: neighbor correlation < 0.8, low SNR, >5% out-of-range samples) and verified visually for drift or disconnection. Rejected channels were reconstructed via fourth-order spherical spline interpolation (29) to preserve spatial continuity of scalp electric fields.(4) Independent component analysis (ICA) was used to identify and remove components reflecting ocular, muscle, and cardiac artifacts.Artifactual components were identified via scalp topography, spectral profile and waveform inspection, cross-validated with the ADJUST toolbox. Ocular (periorbital, δ-dominant, blink-locked), myogenic (frontotemporal, >20 Hz bursts) and cardiac (lateral temporal, heartbeat-synced sharp waves) components were removed, with only definitively non-neural sources excluded.(5) The continuous data were segmented into non-overlapping 2-s epochs, and segments exceeding ± 100 µV were rejected to avoid data redundancy and temporal autocorrelation.(6) Finally, the data were re-referenced to the common average. Preprocessed datasets were subsequently entered into the analyses described below. Epochs with visible baseline drift, electrode popping or non-stationary baseline shifts were also excluded. All epochs were first screened automatically by amplitude thresholding, then manually reviewed and confirmed by a trained researcher to retain only artifact-free segments.

### 2.4 Methods

This pilot trial had prespecified primary outcomes, secondary outcomes, and additional exploratory analyses before data acquisition. Prespecified primary outcomes: Resting-state spectral power across five frequency bands (δ, θ, α, β, γ) and corresponding Group × Time interaction effects. Prespecified secondary outcomes: Scalp topographic distribution of band-limited EEG power. Exploratory analyses: Functional connectivity calculated using the debiased weighted phase lag index (dwPLI), which were not predefined as formal study outcomes.

#### 2.4.1 Power spectral analysis

Power spectral analysis quantifies the distribution of EEG power across canonical frequency bands (δ, θ, α, β, γ), enabling characterization of baseline, task-free brain dynamics and thalamocortical rhythms, and reflecting excitation–inhibition balance and network rhythmic organization (30). To derive resting-state frequency-domain metrics, we estimated the power spectral density (PSD) of preprocessed EEG using Welch’s method (window length 2 s, 50% overlap, Hamming window) over 0.5–40 Hz. Band-limited absolute power was obtained by integrating the PSD within standard bands-δ(0.5–4 Hz),θ (4–8 Hz), α (8–13 Hz), β (13–30 Hz), and γ (30–40 Hz)—and relative power was computed by normalizing to the total power in 0.5–40 Hz. Primary outcomes were band powers at the electrode level and for the predefined scalp regions of interest.

#### 2.4.2 Topographic (scalp map) analysis

Topographic mapping visualizes EEG metrics from scalp electrodes (e.g., band-limited absolute/relative power, ERSPs) by interpolating electrode-wise values according to their spatial locations to yield continuous scalp distributions, thereby depicting spatial organization and hemispheric asymmetries of brain electrical activity (29). After computing PSD within the five predefined frequency bands, band-limited absolute power was obtained by integrating the PSD within each band, and relative power was derived by normalizing to total power in 0.5–40 Hz.

For visualization and statistics, relative band power was standardized via within-subject, band-specific z-scoring across electrodes (i.e., for each participant and band, subtracting the across-electrode mean and dividing by the across-electrode standard deviation). Discrete electrode values were then mapped to a 2D scalp grid using spherical-spline interpolation (e.g., gridscale=256) to render pre-vs post-stimulation topographies and corresponding difference maps. For a given band, a consistent color scale was maintained, and electrode locations were indicated. Non-scalp channels (EOG/ECG) were excluded from plotting. Statistical comparisons at the electrode and region-of-interest levels were conducted on standardized values, with multiple-comparison control (e.g., false discovery rate, FDR).

#### 2.4.3 Exploratory brain network connectivity analysis

From a graph-theoretic perspective, the human brain can be abstracted as a complex network and represented mathematically as a “graph.” Within this framework, connectivity matrices can be transformed into weighted brain networks, enabling quantitative analysis of network properties. In practice, however, raw connectivity matrices often contain numerous weak and noise-driven connections that may obscure biologically meaningful organization and blur key effects. Therefore, prior to network construction, thresholding is commonly applied to sparsify the graph—removing redundant edges while emphasizing connections most informative for analysis (31). In this study, we adopted a data-driven thresholding strategy based on the orthogonal minimum spanning tree (OMST) algorithm (32).

Functional connectivity between channels was estimated with the debiased weighted phase lag index (dwPLI) in FieldTrip across canonical frequency bands. EEG data were processed using 2-second sliding windows with 50% overlap. This configuration enhances sample quantity and signal stability for reliable connectivity estimation. Complex cross-spectra were obtained using a DPSS multitaper approach (mtmfft; tapsmofrq = 2 Hz), from which *dwPLI* was computed. Connectivity was then averaged first across epochs and then within each frequency band to yield individual N×N band-limited connectivity matrices. Group-level matrices were derived by robust averaging across participants.For OMST thresholding, the full symmetric dwPLI matrix was processed per the standard protocol of Dimitriadis et al. (32). The algorithm builds an orthogonal minimum spanning tree backbone, retaining topologically informative edges while removing noise-driven weak connections, with final network density constrained to 10%–15% across all participants.

To highlight the network backbone, strong edges were retained either via OMST-derived sparsification or, for visualization, by applying an additional high-percentile threshold to the upper-triangular edge weights (e.g., 90th–95th percentile) or enforcing an equivalent density (10%–20%), followed by symmetrization. Networks were visualized on a standard equal-angle scalp layout (oriented with Fpz anterior); nodes corresponded to electrodes, and edge color/width varied monotonically with *dwPLI* weight. Statistical assessment of edges at the electrode-and region-of-interest levels used permutation testing with false discovery rate (FDR) correction where appropriate.

For graphical presentation, we selected the top 10% strongest edges to simplify network visualization. It is important to note that this threshold was used exclusively for plotting, and all statistical tests were conducted on the full set of edges without prior thresholding.

#### 2.4.4 Statistical analysis

All statistical analyses were performed using MATLAB R2021b and R 4.2.3 [lmerTest (33), emmeans (34)]. Linear mixed-effects models (LMMs) with Type III Wald chi-square tests and Kenward-Roger df correction were adopted to assess the main effects of Group, Time, and their interaction on log-transformed EEG band power. To control the confounding effects of medications on resting-state EEG rhythms, chlorpromazine equivalent dose and the Anticholinergic Burden Score were included as covariates in all LMMs.

Strict multiple comparison correction strategies were implemented for all EEG statistical tests according to different analytical families. Specifically, FDR correction was used to correct multiple comparisons across five frequency bands (delta, theta, alpha, beta, gamma) for spectral power analysis. For scalp electrode-level topographic analyses, permutation testing combined with FDR correction was performed to control for multiple electrode-wise comparisons. For whole-brain functional connectivity analysis, rigorous non-parametric permutation testing (1000 permutations) coupled with FDR correction was applied to eliminate false positives arising from massive edge-wise pairwise comparisons between brain regions. No ROI-based or multi-time-point comparisons were conducted in this study.

*Post-hoc* comparisons were conducted using estimated marginal means with Bonferroni correction for multiple testing. All effect sizes were reported with standardized indicators, and all tests were two-tailed with a significance level set at α = 0.05.

## 3 Results

### 3.1 Demographic information

**Table 1** presents the demographic characteristics of the study participants. A total of 40 older inpatients aged >60 years with schizophrenia were included, with 20 participants in the PMS group and sham-control group, respectively.

**Table 1:** Demographic and key clinical baseline characteristics of study participants.

Variable	PMS(n=20)	Sham (n=20)	Test result (p)	Standardized mean difference (SMD)
Age (years)	66.55 ± 3.10	65.45 ± 4.55	0.162	0.28
Gender (male: female)	12:08	16:04	0.301	–
PANSS total score	51.00 ± 5.61	48.95 ± 5.14	0.194	0.37
Duration of illness (years)	37.38 ± 9.02	26.00 ± 12.33	0.054	0.78
Number of lifetime episodes	6.50 ± 3.03	6.10 ± 2.90	0.723	0.13
Chlorpromazine equivalent dose (mg/day)	468.63 ± 223.58	401.08 ± 191.69	0.246	0.32
Anticholinergic Burden Score	2.75 ± 1.81	1.88 ± 1.50	0.179	0.52

Data are presented as the mean ± SD for continuous variables, and as a ratio for categorical variables. Continuous variables were analyzed using the Mann–Whitney U test; categorical variables were assessed with Fisher’s exact test.

The groups were comparable in age (66.55 ± 3.10 vs. 65.45 ± 4.55 years; Mann–Whitney U = 252.000, p = 0.162) and sex distribution (male/female: 12/8 vs. 16/4; Fisher’s exact p = 0.301). Baseline symptom severity, indexed by the Positive and Negative Syndrome Scale (PANSS) total score, did not differ between groups (51.00 ± 5.61 vs. 48.95 ± 5.14; U = 236.500, p = 0.194). Illness duration showed a trend toward being longer in the PMS group (37.38 ± 9.02 vs. 26.00 ± 12.33 years; U = 102.000, p = 0.054) but did not reach the significance threshold. The number of lifetime episodes was similar between groups (6.50 ± 3.03 vs. 6.10 ± 2.90; U = 213.500, p = 0.723). Chlorpromazine equivalent dose (468.63 ± 223.58 vs. 401.08 ± 191.69 mg/day; U = 178.500, p = 0.246) and the Anticholinergic Burden Score (2.75 ± 1.81 vs. 1.88 ± 1.50; U = 165.000, p = 0.179) also revealed no significant intergroup differences.

Participants took first- and second-generation antipsychotics, mood stabilizers, cognitive enhancers, and antiparkinsonian medications. Benzodiazepines and extra anticholinergic agents were not prescribed to any participant. All patients kept stable medication regimens with no changes to drug types or dosages in the four weeks prior to intervention. We used chlorpromazine equivalent dose and the Anticholinergic Burden Score to evaluate medication characteristics. Collectively, demographic, clinical, and medication-related variables were well balanced at baseline.

### 3.2 Power spectral analysis

**Figure 2:**
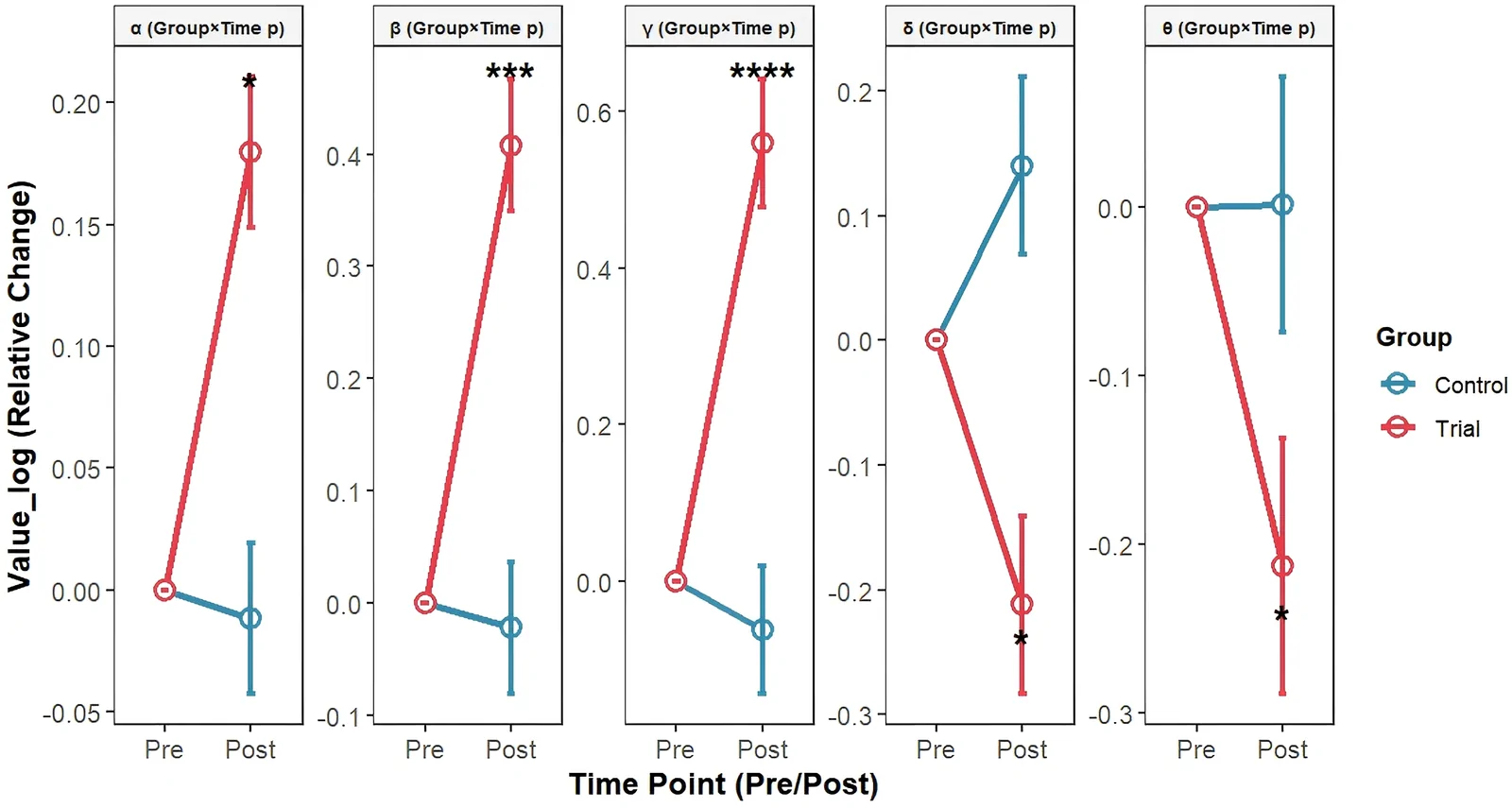
Line plots for Group × Time interaction of EEG band power across five frequency bands. This plot shows relative changes of log-transformed EEG power pre- and post-intervention in two groups. The y-axis represents normalized power for δ, θ, α, β and γ bands. Linear mixed-effects models with FDR correction were used for statistical analysis. * indicates p < 0.05, *** indicates p < 0.001, and **** indicates p < 0.0001.

The frequency band power changes for the two groups are summarized as shown in **Figure 2**. Linear mixed model analysis (fixed effects: group, time, and Group × Time interaction; random intercept for subject) revealed significant Group × Time interactions for all five EEG frequency bands after Benjamini–Hochberg FDR correction across bands (Wald χ²(1) = 4.24–30.55, all q < 0.05),as shown in **Table 2**. Baseline comparisons confirmed no significant between-group differences at pre-intervention in any band (all p > 0.05). Simple effects analysis with Kenward-Roger df correction showed that in the active stimulation group, α (estimate = 0.180, t(42.1) = 5.81, p < 0.001), β (0.408, t(42.1) = 6.93, p < 0.001), and γ (0.560, t(42.1) = 6.86, p < 0.001) power significantly increased post-intervention, while θ (−0.213, t(42.1) = −2.81, p = 0.007) and δ (−0.212, t(42.1) = −2.98, p = 0.005) power significantly decreased; the sham group exhibited no significant within-group changes in any band (all |t| < 0.77, p > 0.45). At post-intervention, the active group showed significantly higher α (estimate = 0.260, t(45.9) = 2.47, p = 0.017), β (0.457, t(54.3) = 3.95, p < 0.001), and γ (0.646, t(58.2) = 4.55, p < 0.001) power, and significantly lower θ (−0.254, t(65.5) = −2.29, p = 0.026) and δ (−0.333, t(54.1) = −2.35, p = 0.023) power versus sham (estimates = active − sham). These results demonstrate that a single session of PMS induces frequency-specific neuromodulation in older adult patients with schizophrenia—elevating high-frequency (α/β/γ) and suppressing low-frequency (θ/δ) oscillations—whereas sham stimulation produced no significant electrophysiological effects.

**Table 2:** Linear mixed-model analysis of oscillatory brain activity: group × time effects.

Band	Group × time interaction χ² (df), P	Control (post−pre) Est (SE), P	Trial (post−pre) Est (SE), P	Between-group (control−trial) pre: Est (SE), P	Between-group (control−trial) post: Est (SE), P
α	20.13 (1), <0.001 ***	−0.012 (0.031), 0.706	0.180 (0.031), <0.001 ***	−0.069 (0.105), 0.515	−0.260 (0.105), 0.017 *
β	28.15 (1), <0.001 ***	−0.022 (0.059), 0.706	0.408 (0.059), <0.001 ***	−0.027 (0.116), 0.820	−0.457 (0.116), <0.001 ***
γ	30.55 (1), <0.001 ***	−0.062 (0.082), 0.449	0.560 (0.082), <0.001 ***	−0.023 (0.142), 0.871	−0.646 (0.142), <0.001 ***
θ	4.24 (1), 0.040 *	0.002 (0.076), 0.980	−0.213 (0.076), 0.007 **	0.039 (0.111), 0.728	0.254 (0.111), 0.026 *
δ	12.85 (1), <0.001 ***	0.140 (0.071), 0.056	−0.212 (0.071), 0.005 **	−0.020 (0.142), 0.888	0.333 (0.142), 0.023 *

Est , Estimate; SE, Standard Error. Control represents the sham group, and Trial represents the active PMS group. Post−Pre indicates the change from pre- to post-intervention. *p < 0.05, **p < 0.01, ***p < 0.001.

**Figure 3:**
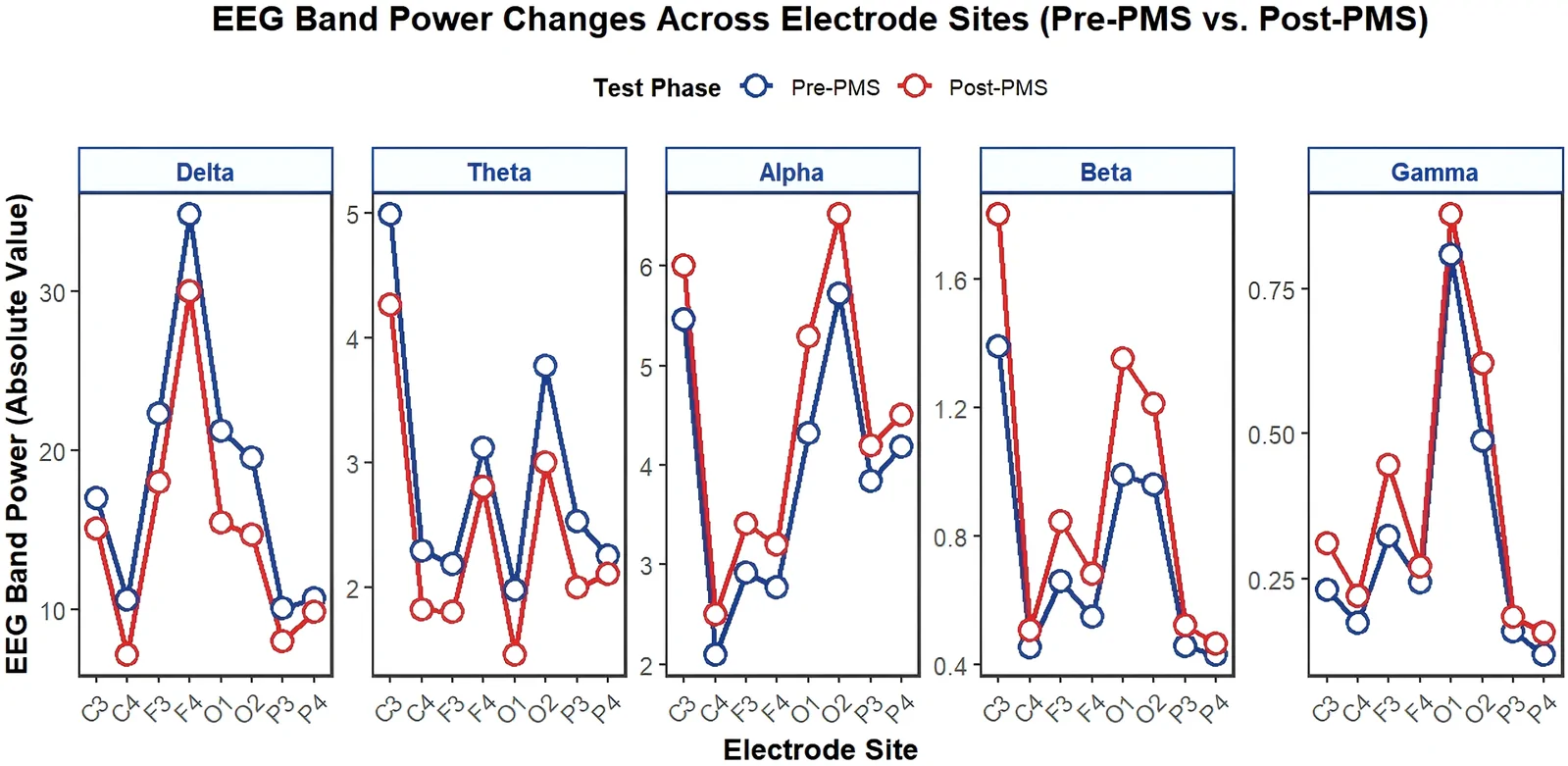
Power spectra of EEG signals at representative scalp electrode sites before and after PMS intervention. Spectral power distributions at eight key electrodes (F3, F4, C3, C4, P3, P4, O1, O2) are shown across δ, θ, α, β and γ bands. All values are absolute spectral power (unit: µV²/Hz). Pre-PMS = recordings before intervention; Post-PMS = recordings within 5 minutes after a single PMS session. Color bar indicates the magnitude of spectral power.

Additionally, we examined pre–post changes in oscillatory power across key scalp sites in the PMS group. **Figure 3** illustrates the power spectra at F3, F4, C3, C4, P3, P4, O1, and O2 before and after stimulation. At baseline, spectral activity was predominantly concentrated in the low-frequency bands (δ, θ). Following PMS, most electrodes exhibited reduced power in low-frequency oscillations accompanied by moderate increases in higher-frequency bands (α, β, γ). Notably, these neuromodulatory effects exhibited clear spatial specificity. Occipital electrodes (O1/O2) showed the most pronounced suppression of low-frequency δ and θ activity. Specifically, δ-band power at the O1 electrode was reduced by 27.3% (effect size d = −1.83, p < 0.001), representing the most prominent decrease among all regions, directly confirming that low-frequency oscillations in the occipital scalp regions are strongly inhibited by PMS; similarly, δ-band power at O2 was decreased by 25.1% (d = −1.67, p < 0.001). In contrast, frontal electrodes (F3/F4) displayed robust enhancement across high-frequency α, β, and γ bands. In particular, γ-band power at F3 was increased by 37.8% (d = 1.67, p < 0.001), supporting the observation that high-frequency oscillations indicate that neural activity at frontal scalp regions is preferentially modulated by PMS. The parietal region (P3/P4) showed a relatively modest response, with significant enhancement limited mainly to the γ band (increases of 16.1% and 32.4% at P3 and P4, respectively), suggesting a weaker modulatory effect of PMS in this region. The central region (C3/C4) exhibited a mixed pattern of “low-frequency inhibition plus mild high-frequency enhancement,” with a spatial profile intermediate between the frontal and occipital areas, further supporting the region-specific modulation of oscillations across distinct scalp regions by PMS. Oscillations by PMS. Collectively, these findings confirm that PMS exerts a targeted regulatory effect on scalp-level electrophysiology, with region-specific modulation of low- and high-frequency oscillations, providing direct electrophysiological evidence for its neuromodulatory mechanism.

### 3.3 Topographic map analysis

**Figure 4:**
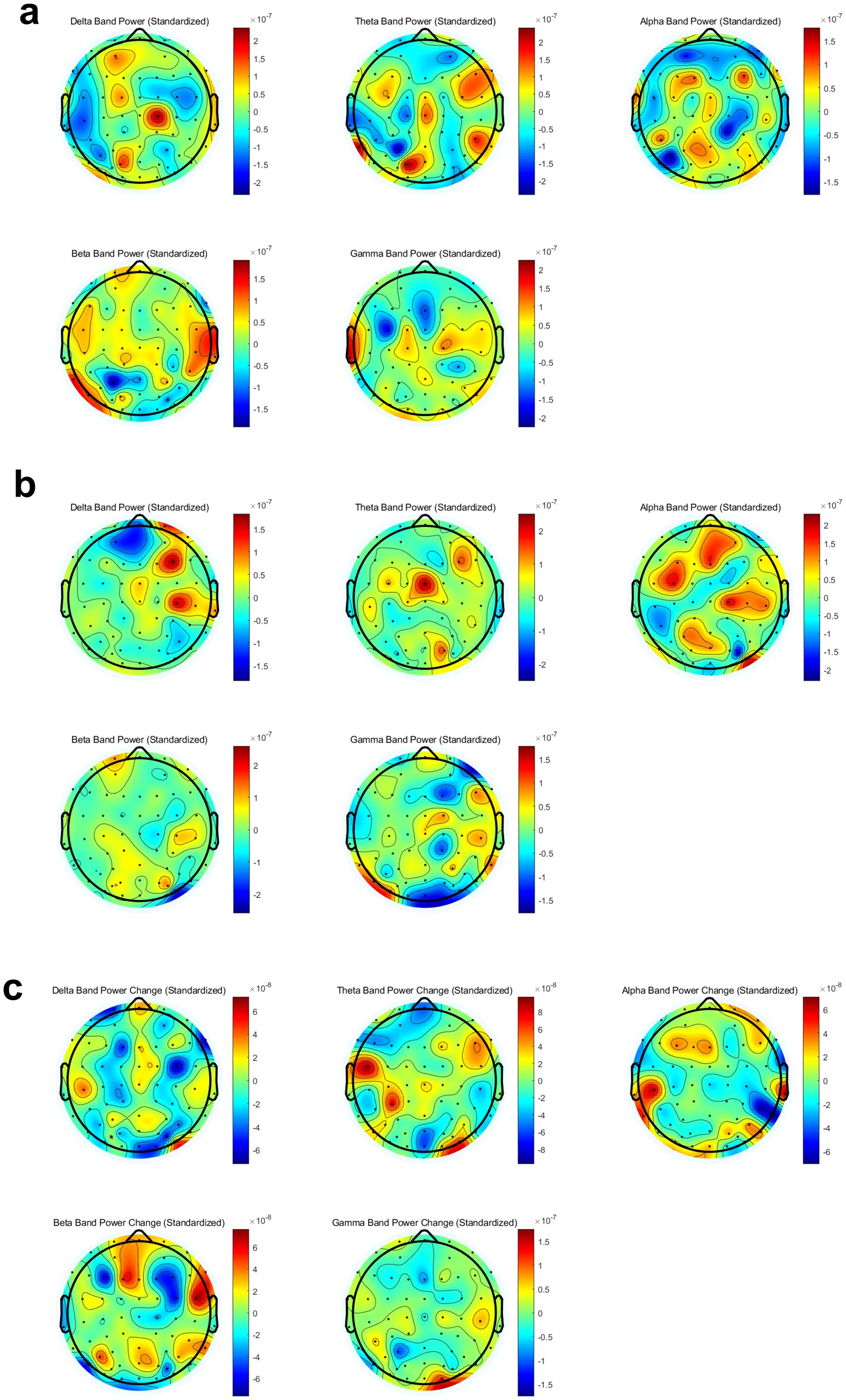
Topographic distributions of standardized band power across five frequency bands. **(a)** Topographic maps of band power at baseline (pre-intervention); **(b)** Topographic maps after PMS intervention; **(c)** Difference maps (post minus pre) showing power changes. All values are z-standardized relative band power. Color bars denote the range of standardized values. Statistical comparisons were conducted using permutation testing combined with FDR correction; *p < 0.05, **p < 0.01.

The topographic spatial distributions of band-limited power (δ, θ, α, β, γ) are illustrated in **Figure 4**. In the pre-PMS topographic maps (Panel a), the normalized power of each frequency band exhibited a distinct spatial distribution pattern. Specifically, the δ band showed relatively higher power predominantly in the central regions (Cz, C3, C4) and bilateral temporal areas (T3, T4), with lower power concentrated at scalp margins, including the occipital regions (O1, O2) and frontopolar electrodes (Fp1, Fp2). The θ band presented high power in discrete patches over the right frontotemporal region (F4, T4) and left central area (C3), accompanied by obvious low-power zones in the left temporoparietal region (T3, P3) and inferior central scalp. The α band displayed relatively elevated power in the central region (Cz) and right parietotemporal area (P4, T4), whereas reduced power was observed in the left frontal region (F3) and midline central area. For the β band, high power was mainly concentrated in the right frontoparietal region (F4, P4) and inferior temporal area (T4), in sharp contrast to the low power in the left central region (C3). The γ band exhibited higher power along the left temporooccipital margin (T3, O1) and partial central scalp (Cz, C3), while low power predominated in the vertex midline (Fz, Cz, Pz) and right parietal region (P4).

In the post-PMS topographic maps (Panel b), marked spatial reorganization was observed in the power distribution of each frequency band. The δ band showed significantly increased power in the right frontoparietal region (F4, P4) and decreased power in the left superior frontoparietal area (F3, P3), with an overall more diffuse distribution. The θ band formed a prominent central core of high power across the central regions (Cz, C3, C4), with low power diffusely distributed over peripheral scalp areas such as bilateral temporooccipital regions. The α band exhibited multiple extensive high-power zones in bilateral parietooccipital regions (P3, P4, O1, O2) and the right frontal region (F4), presenting a wider overall distribution with relatively scattered low-power areas mainly localized near the left frontal region (F3). The β band showed a widespread power distribution characterized by an overall moderate-to-high pattern, with only a few localized low-power patches in the left temporal region (T3). For the γ band, high power was mainly distributed at the temporooccipital margins (T3, T4, O1, O2) and the right frontal region (F4), while multiple low-power foci emerged over the central scalp (Cz, C3), indicating distinct spatial differentiation.

In the FDR-corrected post- vs. pre-PMS spatial contrast maps (Panel c), power changes demonstrated significant band specificity, with overall power reductions in the δ and θ bands and increases in the α, β, and γ bands. Specifically, the δ band exhibited extensive blue coloration over the vertex midline (Fz, Cz, Pz) and right frontocentral region (F4, C4) (p < 0.01), indicating significantly lower power post-stimulation relative to baseline, with only a few small regions of increased power visible in the left temporal area (T3). The θ band was dominated by blue coloration in the left temporoparietal region (T3, P3) and inferior central scalp (p < 0.05), also reflecting a significant post-stimulation power reduction. In contrast, the α band displayed extensive and concentrated red coloration over the superior parietal region (Pz, P3, P4) and bilateral temporooccipital margins (T5, T6, O1, O2) (p < 0.05), indicating a marked post-stimulation power increase. The β band showed prominent red coloration across the central regions (Cz, C3, C4) and the right frontoparietal area (F4, P4) (p < 0.01), with only minor low-power regions in the left frontal area (F3). The γ band was mainly characterized by red coloration along the peripheral scalp margins (T3, T4, O1, O2) and partial midline electrodes (Fz, Pz) (p < 0.01), with sparse power reduction limited to the left occipital region (O1). This band-specific modulation pattern indicates that PMS exerts differential regulatory effects on EEG activity, manifested as decreased power in slow-wave bands (δ, θ) and increased power in fast-wave bands (α, β, γ).

### 3.4 Brain network analysis

**Figure 5:**
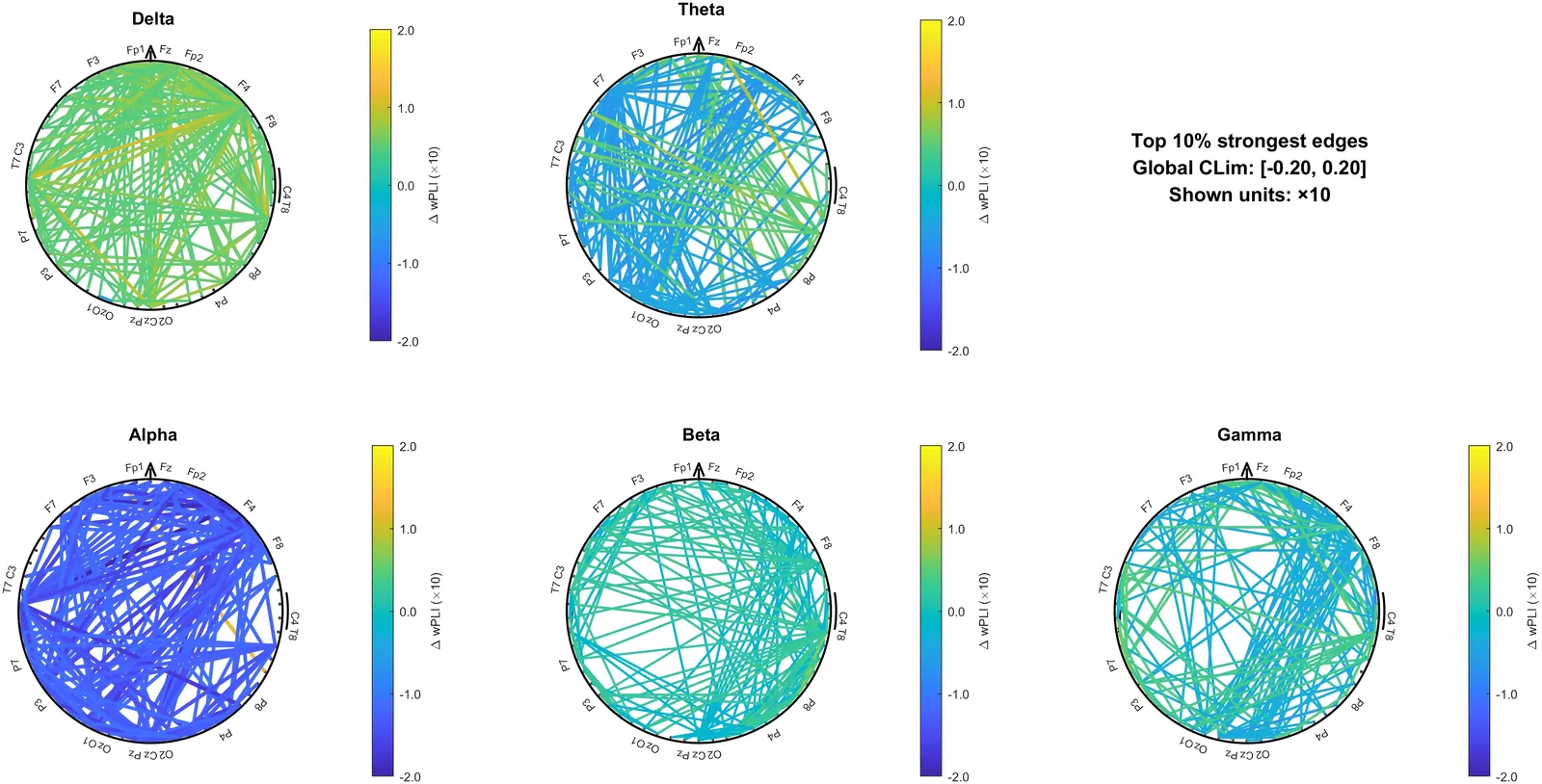
Changes in debiased weighted phase lag index (dwPLI) after PMS intervention.All dwPLI connectivity analyses shown here are exploratory and not counted as prespecified primary or secondary outcomes. Group-level ΔdwPLI (post minus pre) across canonical EEG bands. Top 10% edges are retained for visual presentation exclusively. Statistical analyses of all edges adopted 1000 permutations with FDR correction. Nodes correspond to 59 scalp electrodes; edge features encode ΔdwPLI. Global limit: [−0.20, 0.20]. Values multiplied by 10 for visualization.

Compared with the pre-test, the group-mean difference in debiased wPLI (**Figure 5**; values shown on the display scale, ΔwPLI × 10) exhibited a clear, band-specific enhancement pattern. Overall, the low-frequency bands were characterized primarily by strengthened prefrontal–central coupling and anterior–posterior integration, whereas the higher-frequency bands progressively shifted toward locally enhanced synchrony within prefrontal and lateral parietal regions. Specifically: δ(1–4 Hz): Predominantly increased coupling between the right prefrontal and central regions; representative pathways included FC2–C2(1.15), FC2–C1(1.03), FCz–Cz(0.89), and Cz–CP2(0.79).θ(4–8 Hz): Enhanced long-range coupling between the frontal vertex/midline and the right posterior scalp regions, together with increased intra-network synchrony within the right temporo-parietal association area; representative pathways included FCz–C2(0.93), FC5–TP8(0.73), Fpz–P4(0.68), and TP8–P8(0.61). α(8–13 Hz): Strengthened cross-hemispheric integration between frontal and parietal regions, accompanied by coordinated increases over the midline occipital cortex; representative pathways included AF3–CP6(1.20), FC5–CP6(1.02), FCz–Oz(0.84), and AF3–P6(0.81). β(13–30 Hz): Increases mainly concentrated within the right temporo-parietal network, with a small number of cross-hemispheric links projecting from the left frontal to the right temporal regions; representative pathways included T8–P8(0.68), TP8–P8(0.58), F1–T8(0.44), and FC4–FC6(0.40). γ(30–40 Hz): Predominantly local or short-range synchrony enhancement involving the frontopolar/anterior frontal cortex and the lateral parietal cortex; representative pathways included Fpz–F2(0.64), FC4–FC6(0.57), CP5–P7(0.56), and T8–P8(0.49).

### 3.5 Safety and tolerability

No participants withdrew, and no severe adverse events were reported during the intervention. However, routine blood pressure and heart rate were not tracked, so conclusions regarding comprehensive cardiovascular safety cannot be drawn. Subjective tolerability appeared favorable within this limited observation window.

## 4 Discussion

This exploratory neurophysiological pilot study sought to preliminarily map transient resting-state EEG alterations triggered by one session of PMS in older adults with schizophrenia, including frequency-specific spectral shifts, scalp topographic reorganization, and modified interregional functional coupling. The observed band-specific electrophysiological patterns constitute exploratory observational findings rather than confirmatory evidence for therapeutic action.

### 4.1 Significance of spectral changes

Following PMS, downregulation of low-frequency power (δ, θ) together with upregulation in the mid-to-high frequencies (α, β, γ) may indicate a shift of resting-state cortical rhythms toward a “faster, more fine-grained”encoding regime (35). Low-frequency rhythms are typically associated with large-scale, low-resolution integrative activity and scalp-level inhibitory activity; their relative amplification is often regarded as a pathophysiological hallmark of resting-state schizophrenia (36–38). In this study, reductions in δ and θ power reflect a measurable shift away from the slow-wave dominant oscillatory pattern observed at baseline (39). Correspondingly, α increases are widely interpreted as restoration of thalamo–cortical rhythmicity and optimization of sensory–cognitive gating (40), whereas elevations in β and γ are commonly linked to rebalancing of excitation–inhibition at corresponding scalp regions and enhanced sensorimotor and higher-order cognitive processing (41, 42).

At the electrode level (e.g., low-frequency reductions at F3, C3, and O1 and high-frequency increases at P3 and C4), PMS produced directionally consistent—though variably scaled—modulations across prefrontal, central, and occipital sites. This pattern aligns with ascending proprioceptive drive along the spino–cerebello–thalamo–cortical pathways (43), which preferentially engage sensorimotor and parieto-occipital channels (44). Overall, a single PMS session produced a measurable shift toward faster oscillatory activity across scalp electrodes (45), reflecting altered resting-state rhythmic patterns in this exploratory sample.

### 4.2 Spatial distribution and neural mechanisms

Our topographic results indicate clear regional specificity after PMS: δ/θ power decreased broadly over superior, left, and inferior scalp regions; α power increased prominently over superior areas and along the bilateral peripheral rim; and β/γ power rose mainly over central, right-hemispheric, and peripheral regions. In focal territories, decreases in δ/θ suggest attenuation of a slow-wave–dominated, low-efficiency functional state, effectively reducing cortical “background noise” and thereby improving information-processing efficiency (46). Consistent with the Jensen–Mazaheri account of alpha as inhibitory gating (44), and Klimesch’s inhibition–timing framework, enhanced α over occipital and parietal scalp regions would suppress responses to irrelevant input while stabilizing local rhythmic activity—akin to cleaning the sensory backdrop for vision and attention, yielding more precise perception and tighter focus (47).

For θ, in line with the Cavanagh–Frank view of midfrontal theta as a domain-general control signal and the Lisman–Jensen θ–γnesting framework, the concurrent downshifts of θ and δ at rest are best interpreted as suppression of nonspecific, diffuse slow-wave components while preserving functional reserves in control- and memory-related channels. This pattern can reduce redundant synchrony and stabilize network thresholds (48, 49).

Regarding γ, increases in γ synchrony support rapid feedforward transmission and local feature binding under Buzsáki’s local computation/feature-binding account and Fries’s communication-through-coherence framework (50, 51). Together with frequency-specific division of labor proposed by Bastos and Michalareas (γfavoring feedforward, β favoring feedback), the observed co-elevation of γ and β over central and right-hemispheric regions align with the demands of sensorimotor and lateral parietal circuits for rapid encoding, state maintenance, and cross-feature integration (52, 53).

### 4.3 Changes in functional connectivity and clinical implications

The *dwPLI* difference maps revealed a dual specificity at the band–network level. δ showed strengthened right prefrontal–central coupling, consistent with an optimization characterized by less slow-wave power but greater phase consistency—i.e., a convergence from diffuse slow-wave activity toward a “necessary low-frequency backbone” that supports basic homeostasis and motor readiness (36, 54).θ exhibited increased long-range coupling from the midline frontal vertex to the right posterior cortex, alongside heightened intra-network synchrony within the right temporo-parietal association zone. Given that θ-mediated long-range links are commonly associated with cognitive control, error monitoring, and cross-modal integration,these band-specific coupling shifts reflect altered functional coordination within the fronto–parietal network (48). α showed cross-hemispheric frontal–parietal integration with co-elevation over the midline occipital cortex, aligning with α-coupling’s role as a “network gate” for sensory suppression and attentional control, potentially reducing the intrusion of irrelevant internal and external information (44). β increased within the right temporo-parietal network with a few interhemispheric links; because β-coupling relates to sensorimotor integration, predictive coding, and state maintenance, these changes suggest stabilization of the sensorimotor framework (55). γ displayed enhanced short-range synchrony over frontopolar/anterior frontal and lateral parietal regions. γ is often viewed as a readout of local feature binding and rapid information integration, and its elevation here may correspond to immediate gains in perception and working memory (50, 56).

Clinically, These band-specific connectivity shifts represent exploratory electrophysiological observations linked to oscillatory coordination. Future larger trials incorporating formal clinical assessments are required to examine whether sustained PMS-related EEG shifts correlate with behavioral or symptomatic measures in older adults with schizophrenia (45, 57).

### 4.4 Comparison with established rTMS literature

As a pilot investigation, this study preliminarily observed that a single session of PMS exerts a measurable modulatory effect on resting-state EEG oscillations in older adults with schizophrenia. Within the broader framework of non-invasive brain stimulation for cognitive regulation, this finding shares conceptual consistency with relevant conclusions from repetitive transcranial magnetic stimulation (rTMS) research (58).

As a well-investigated magnetic stimulation modality, rTMS targeting the left dorsolateral prefrontal cortex (DLPFC) has been reported to induce neurocognitive enhancement in multiple studies, serving as a classic reference paradigm for magnetic stimulation-based neuromodulation research (59–61). In terms of electrophysiological response patterns, the trend of reduced δ/θ low-frequency power and elevated α/β/γ high-frequency power following PMS shows parallels to time-frequency EEG findings of rTMS in psychiatric populations. This consistent direction of change implies that magnetic stimulation delivered via distinct pathways may share overlapping electrophysiological response mechanisms, though such cross-modality correspondence remains to be validated in controlled studies (62, 63). Research on rTMS in older adults with memory dysfunction also indicates that magnetic stimulation can influence cognitive performance by modulating cerebral network oscillations, providing supportive evidence for the feasibility of magnetic neuromodulation in the aging brain (64).

The two modalities differ in mechanistic pathways and practical properties. rTMS directly targets intracranial cortex with higher spatial focality, and its reported regulatory effects are relatively more definitive in existing literature. By contrast, PMS acts on the central nervous system indirectly through ascending peripheral pathways; while less focal, it offers better portability, lower operational barriers, and favorable tolerability, making it more suitable for bedside and long-term care settings. Methodologically, research frameworks from the rTMS field, including treatment response prediction based on nonlinear EEG features and machine learning, as well as combined task-stimulus paradigms, can provide valuable references for future PMS studies on parameter optimization and beneficiary stratification (65–68).

Overall, PMS may serve as a potential adjunctive intervention candidate for older adults with schizophrenia. Magnetic stimulation has also shown application potential in other neurological conditions such as epilepsy (69–72), indicating broad exploratory space for PMS. Its specific effect profiles and clinical positioning still require further clarification through standardized head-to-head controlled studies.

### 4.5 Limitations and future directions

This was a single-center study with a small sample, a single stimulation session, and with immediate post-session assessment. Furthermore, this study only collected baseline PANSS scores for between-group baseline matching. No post-intervention PANSS assessments, cognitive and attention measurements, or patient-reported outcomes were performed. The absence of these clinical outcome data limits our ability to evaluate the clinical efficacy and overall safety of PMS. We will incorporate these indicators and corresponding analyses in future studies. Moreover, EEG analyses centered on spectral power and static connectivity offer a relatively narrow metric set and lack more direct source-level neurophysiological evidence. In addition, we did not monitor vital signs including blood pressure and heart rate in the present study. Future trials will add continuous vital sign monitoring and standardized safety assessments to comprehensively evaluate the safety and tolerability of PMS.

Although medication status was well balanced at baseline, and we controlled for confounders by including chlorpromazine equivalent dose and the Anticholinergic Burden Score as covariates, psychotropic drugs may still affect resting-state EEG in older adults with schizophrenia, and we will standardize medication regimens in future studies to minimize such interference. We also lacked continuous vital sign monitoring including blood pressure and heart rate, restricting our ability to fully characterize the cardiovascular safety profile of PMS.Future work requires multicenter, double-blind randomized trials with longitudinal follow-up to compare PMS stimulation parameters and verify their long-term efficacy and safety. Meanwhile, patients’ experience, usability, and acceptability of PMS should be assessed to optimize clinical application and treatment outcomes.

## 5 Conclusion

This study provides the first evidence that a single session of PMS can elicit changes in resting-state EEG in older adults with schizophrenia. We observed decreases in low-frequency power (δ, θ) alongside increases in mid–high frequencies (α, β, γ), with a localized spatial pattern characterized by occipito–parietal α enhancement and central/right-lateralized β and γ up shifts, accompanied by band-specific dwPLI connectivity shifts. No comparable changes were observed in the sham-stimulation group.

Taken together, these findings suggest that PMS can, within minutes, induce distinct band-specific oscillatory shifts, including reduced low-frequency power and elevated mid-to-high frequency synchrony across scalp networks. The functional significance of these EEG alterations remains to be tested in future trials incorporating formal cognitive and clinical outcome assessments. As a non-invasive, bedside-feasible intervention, PMS exhibits suggestive preliminary neurophysiological signals in this exploratory pilot dataset; definitive clinical utility must be verified in larger confirmatory neurophysiology trials.Confirmation in multicenter, double-blind randomized trials with longitudinal follow-up is warranted to establish reproducibility, durability, and links to clinical outcomes, and to optimize individualized stimulation parameters and synergistic combinations with rehabilitation or cognitive training.

## Data Availability

The raw data supporting the conclusions of this article will be made available by the authors, without undue reservation.
